# Knowledge of HIV Status Is Associated With a Decrease in the Severity of Depressive Symptoms Among Female Sex Workers in Uganda and Zambia

**DOI:** 10.1097/QAI.0000000000002224

**Published:** 2019-10-16

**Authors:** Katrina F. Ortblad, Daniel Kibuuka Musoke, Michael M. Chanda, Thomson Ngabirano, Jennifer Velloza, Jessica E. Haberer, Margaret McConnell, Catherine E. Oldenburg, Till Bärnighausen

**Affiliations:** a Department of Global Health, University of Washington, Seattle, WA;; b International Research Consortium, Kampala, Uganda;; c John Snow, Inc., Lusaka, Zambia;; d Uganda Health Marketing Group, Kampala, Uganda;; e Massachusetts General Hospital Global Health, Boston, MA;; f Department of Global Health and Population, Harvard T.H. Chan School of Public Health, Boston, MA;; g Francis I. Proctor Foundation, University of California San Francisco, San Francisco, CA;; h Department of Ophthalmology, University of California, San Francisco, San Francisco, CA;; i Department of Epidemiology & Biostatistics, University of California, San Francisco, CA;; j Africa Health Research Institute (AHRI), KwaZulu-Natal, South Africa; and; k Heidelberg Institute of Public Health, Heidelberg University, Heidelberg, Germany.

**Keywords:** HIV status knowledge, HIV testing, depressive symptoms, female sex workers, Uganda, Zambia

## Abstract

Knowledge of HIV-positive status may result in depressive symptoms, which may be a concern to scaling novel HIV testing interventions that move testing outside the health system and away from counselor support.

**Setting:**

Uganda and Zambia.

**Methods:**

We used longitudinal data from 2 female sex worker (FSW) cohorts in Uganda (n = 960) and Zambia (n = 965). Over 4 months, participants had ample opportunity to HIV testing using standard-of-care services or self-tests. At baseline and 4 months, we measured participants' perceived knowledge of HIV status, severity of depressive symptoms (continuous PHQ-9 scale, 0–27 points), and prevalence of likely depression (PHQ-9 scores ≥10). We estimated associations using individual fixed-effects estimation.

**Results:**

Compared with unknown HIV status, knowledge of HIV-negative status was significantly associated with a decrease in depressive symptoms of 1.06 points in Uganda (95% CI −1.79 to −0.34) and 1.68 points in Zambia (95% CI −2.70 to −0.62). Knowledge of HIV-positive status was significantly associated with a decrease in depressive symptoms of 1.01 points in Uganda (95% CI −1.82 to −0.20) and 1.98 points in Zambia (95% CI −3.09 to −0.88). The prevalence of likely depression was not associated with knowledge of HIV status in Uganda but was associated with a 14.1% decrease with knowledge of HIV-negative status (95% CI −22.1% to −6.0%) and a 14.3% decrease with knowledge of HIV-positive status (95% CI −23.9% to −4.5%) in Zambia.

**Conclusions:**

Knowledge of HIV status, be it positive or negative, was significantly associated with a decrease in depressive symptoms in 2 FSW populations. The expansion of HIV testing programs may have mental health benefits for FSWs.

## Abstract 2

Supplemental Digital Content is Available in the Text.

## BACKGROUND

Female sex workers (FSWs) are at increased risk of HIV infection compared with women of reproductive age in the general population.^1,2^ In low- and middle-income countries, FSWs are 13 times more likely to be living with HIV compared with other women.^1^ FSWs are at increased risk of HIV infection as a result of multiple sexual partners, inconsistent condom use, increased prevalence of physical and sexual violence, and punitive legal systems.^2^ For these reasons, FSWs are encouraged to test for HIV frequently to detect breakthrough infections. Recent studies have shown that novel technologies such as HIV self-testing, which moves HIV testing outside of the controlled environment of the health care system,^3,4,5,6,7^ can significantly increase frequent HIV testing among FSWs.^3^ However, concerns remain that decoupling testing from counseling with this new HIV testing technology might increase depressive symptoms and suicidal ideation among FSWs who test HIV positive.^8,9^

These concerns come from previous studies, conducted among diverse populations, which show that HIV infection is associated with greater depressive symptom severity and risk of major depressive disorder.^10,11,12,13,14,15,16,17^ In sub-Saharan Africa (SSA), the prevalence of likely depression is approximately 2 times greater among individuals living with HIV compared with those in the general population.^18^ The majority of these studies, however, used cross-sectional data or focused on individuals already living with HIV, thus making it difficult to determine whether new knowledge of HIV infection caused depressive symptoms or whether depressive symptoms preceded HIV infection. In other studies, the prevalence of likely depression has been associated with increased sexual risk-taking behaviors among both individuals living and not living with HIV.^19,20,21,22,23^

Understanding how changes in knowledge of HIV status affect the severity of depressive symptoms is important for understanding how to develop and deliver HIV testing interventions that will enhance the well-being, social functioning, and safety of FSW populations. With this analysis, we aimed to understand the association between changes in knowledge of HIV status and the severity of depressive symptoms among FSWs in Uganda and Zambia. In this study, we used changes in FSWs' perceived knowledge of HIV status rather than their actual HIV status because this more directly affects symptoms of depression. In addition, we used cohort data and fixed-effects estimation, controlling for all—that is, observed and unobserved—individual-level confounding factors that did not vary over the observation period (eg, genetic makeup, stable psychological traits, and ethnic, religious, and social backgrounds) as well as all confounding factors that did not vary across individuals. This approach increases the strength of causal inference over and above that of previous studies that could not control for unobserved confounding factors.^24,25^ Based on the previous literature,^10,11,12,13,14,15,16,17^ we hypothesized new knowledge of HIV-positive status (often acquired from HIV testing in the past 1 to 4 months) to be associated with an increase in the severity of depressive symptoms and prevalence of likely depression among FSWs in these high HIV prevalence settings.

## METHODS

### Study Design

We used longitudinal cohort data and an individual fixed-effects estimation approach to measure associations between knowledge of HIV status and depressive symptoms among Ugandan and Zambian FSWs. The FSW cohorts used in this study were formed as part of 2 3-arm cluster-randomized controlled trials testing 2 different peer-based HIV self-testing delivery models conducted in urban Uganda from October 2016 to March 2017 (ClinicalTrials.gov: NCT02846402)^3^ and 3 Zambian transit towns from September 2016 to April 2017 (ClinicalTrials.gov: NCT02827240).^4^ The trials followed identical protocols and measured identical primary outcomes: any HIV testing at 1 and 4 months.

In these trials, participants were randomized in groups of 1 peer educator and 8 participants (Uganda)^3^ or 6 participants (Zambia)^4^ to one of 3 study arms: (1) direct provision of an HIV self-test from a peer educator; (2) facility collection of an HIV self-test, upon presentation of a coupon given to participants by a peer educator; or (3) referral to standard-of-care HIV testing services by a peer educator. All participants completed 4 peer educator visits over the 4-month duration of the trials; the first was a group visit (at month 0), and the subsequent 3 were individual visits (at months 0.5, 1.5, and 3). At each visit, peer educators distributed condoms, information about HIV protection, and encouraged participants to test for HIV. Participants in the HIV self-testing interventions arms additionally received from their peer educator 1 HIV self-test or a coupon exchangeable for an HIV self-test at local health facilities at the first (month 0) and third (month 3) peer educator visit.

Ethical approval for the trials was granted by the Mildmay Uganda Research Ethics Committee (Kampala, Uganda), the Excellence in Research Ethics and Science (ERES) Converge institutional review board (Lusaka, Zambia), and the Harvard T.H. Chan School of Public Health (Boston, USA). Written informed consent was obtained from all participants.

### Study Setting

The randomized trials from which the FSW cohorts used in this study took place in 2 settings: (1) urban Uganda and (2) 3 Zambian transit towns. The Uganda trial took place in Kampala, the capitol city. Kampala is a dense urban center with approximately 13,000 FSWs living there, 1 in 3 of which is estimated to be living with HIV.^26^ Although sex work is illegal in Uganda, a number of free FSW-friendly services (including family planning and HIV testing) are available to Kampala-based FSWs through the Ugandan Ministry of Health's Most At-Risk Population Initiative (MARPI). The Zambia trial took place in Livingstone, Chirudu, and Kapiri Mposhi—transit towns where commercial trucks are often delayed for several hours or days at either border crossings or weight stations.^27^ One in 2 FSWs in Zambia is estimated to be living with HIV.^28^ At the time of the study, there were no free FSW-friendly services available to FSWs working in these Zambian transit towns.

### Participants

In both FSW cohorts, participants were eligible for trial participation if they were older than 18 years, exchanged money or goods for sex in the past month, had not recently tested for HIV (<3 months), and tested HIV-negative at their last test or did not know their HIV status.^3,4^ Participants were ineligible for participation if they did not meet all the previously described criteria. Potential participants were first screened over the phone and then invited for in-person eligibility assessment. FSWs trained as peer educators recruited all trial participants from their existing social networks. These peer educators were referred to study staff by local FSW nongovernmental organizations working in the study settings and from staff at MARPI health care facilities in Kampala. There were no educational or age requirements for FSW peer educators; trust and respect within the local FSW communities were the most important prerequisites.^3,4^

In Uganda, 1587 potential participants were recruited for participation, of which 960 (60%) were enrolled and randomized; in Zambia, 1290 potential participants were recruited for participation, of which 965 (75%) were enrolled and randomized. Some of the most common reasons for nonparticipation in both settings were self-reported HIV-positive status and recent HIV testing (<3 months). Loss to follow-up at 4 months was low in both study settings: only 10% (n = 99) of participants in Uganda and 7% (n = 67) of participants in Zambia were not reached for the final round of data collection. By the end of both HIV self-testing trials, almost all participants in Uganda (95%) and Zambia (96%) had tested for HIV in the past 4 months.^3,4^

### Depressive Symptoms Outcome

We measured depressive symptoms using the Patient Health Questionnaire-9 item (PHQ-9) depression scale.^29^ The PHQ-9 is a screening tool for depression that includes items on somatic depressive symptoms (eg, trouble eating or sleeping), cognitive symptoms (eg, little interest or pleasure in things), and suicidal ideation.^29^ Each of the 9 items is scored on a Likert scale from 0 (“not at all”) to 3 (“nearly every day”), and the sum score provides a continuous measure of depressive symptom severity (range: 0–27), with higher scores indicating greater symptom severity.^29^ For this study, we measured depressive symptoms in 2 ways: (1) the severity of depressive symptoms using a continuous PHQ-9 score and (2) the prevalence of likely depression by constructing a binary variable using a cutoff score ≥10. Our cutoff score for likely depression has been previously validated among women in SSA and corresponds well with a clinical diagnosis of depression.^13,30,31,32,33,34,35^ In addition, the Cronbach's alpha statistic for the PHQ-9 scale was >0.8 at all measurements in both Uganda and Zambia, signaling the reliability of the scale in these FSW cohorts.^36^

Given concerns about the associations between HIV status and suicidal ideation, intent, and behavior,^8,9^ we also looked separately at the proportion of patients reporting suicidal ideation on the PHQ-9. We categorized patients as having suicidal thoughts if they reported thinking they “would be better off dead” or hurting themselves in some way on at least several days in the past 2 weeks.

### Knowledge of HIV Status Predictor

We categorized participants' knowledge of HIV status into 3 states: (1) knowledge of HIV-negative status, (2) unknown HIV status, and (3) knowledge of HIV-positive status. When measuring participants' knowledge of HIV status, we wanted their most up-to-date knowledge of HIV status, captured in their perceived likelihood of being HIV infected. At each round of data collection, we asked participants to estimate the likelihood they were currently living with HIV on a 1–10 ladder scale.^37^ We categorized rungs 1–3 as knowledge of HIV-negative status, rungs 4–7 as knowledge of unknown HIV status, and rungs 8–10 as knowledge of HIV-positive status.

We chose this measurement for knowledge of HIV status because it is the perception of HIV status that will primarily affect depressive symptoms. Participants' perceived knowledge of HIV status at the time of data collection might differ from the results of their last HIV test for reasons including a recent HIV risk encounter after HIV testing, mistrust in a new HIV testing technology (ie, HIV self-testing), or an outside intervention (ie, religious encounter) that may have “cured” the participant of HIV.^38,39^ This measurement of knowledge of HIV status was adapted from a study that measured participants' perceived risk of HIV acquisition^37^ and was used in a similar study that explored the association between knowledge of HIV status and HIV risk-related sexual behaviors among the same cohort of Ugandan FSWs.^40^

### Covariates

We measured sociodemographic characteristics for all participants, including age, education, and income. In addition, participants were asked to report when they last tested for HIV and their sexual behaviors with clients. If participants reported not using condoms with at least 1 client on an average working night, their condom use was categorized as inconsistent. The round of data collection and calendar month was automatically captured in our electronic data collection platform.

### Procedures

Participants completed 3 rounds of data collection: at baseline before the first peer educator visit and then at 1 month and 4 months after the first peer educator visit. At each round, research assistants conducted quantitative questionnaires in face-to-face interviews at a location selected by the study participant (usually an empty bar, home, or guest house). Questionnaires were conducted in the participants' preferred language (English, Luganda Nyanja, Bemba, or Tonga). All data were collected electronically on tablets using CommCare (Dimagi Inc., Cambridge, MA).^3,4^

### Statistical Analysis

We used linear probability models (LPMs) with individual fixed effects to measure the association between participants' knowledge of HIV status and their severity of depressive symptoms (continuous PHQ-9 score), prevalence of likely depression (PHQ-9 scores ≥10), and suicidal ideation (binary outcome). We used individual longitudinal data from the baseline and 4-month assessments from both FSW cohorts, including control and intervention participants together. For our binary outcomes, we chose LPM over logit regression models because the coefficients in LPM are percentage point changes, which are easy to interpret. Individual fixed-effects panel estimation controls for any confounders at the level of the individual that are constant over the observation period.^24,41^ To control for confounders that vary across round or time but are constant across individuals, we further added fixed effects for round of data collection and for calendar month. We adjusted our standard errors for clustering at the level of the peer educator because peers were used to recruit study participants and deliver study interventions. We separated the analyses that used Uganda and Zambia cohort data because of substantial differences in the demographics of and access to free health services for these FSW populations.^3,4^ We used Stata 15.1 (StataCorp, College Station, TX) for all analysis and a *P* value of 0.05 to measure significance.

### Sensitivity Analyses

We conducted 3 sensitivity analyses. First, we tested the robustness of the LPMs with Poisson regression models for the severity of depressive symptoms' outcome and logit regression models for the prevalence of likely depression outcome. As with the primary analyses, these models included individual-level fixed effects, controlled for round of data collection and survey month, and clustered standard errors at the level of the peer educator. Second, we limited our sample to participants who reported testing for HIV since the start of both trials to understand the relationship between knowledge of HIV status and depression among those whose knowledge of HIV status likely changed as a result of recent HIV testing. We calculated effect size estimates using the LPMs with individual fixed effects described above. Third, we used the same model as that used in the primary analyses but additionally adjusted for participants' sexual risk behaviors, specifically their inconsistent condom use with clients.

### Subgroup Analyses

We conducted 2 subgroup analyses. First, to understand the effect of preexisting depressive symptoms on the association between knowledge of HIV status and depressive symptoms, we split participants again into 2 groups based on those who were likely depressed (PHQ-9 scores ≥10) and not likely depressed (PHQ-9 scores <10) at baseline. Second, to understand the effect of access to HIV self-testing on the association between knowledge of HIV status and depressive symptoms, we split participants into 2 groups based on whether they were randomized to one of the HIV self-testing intervention arms or referred to standard-of-care HIV testing services. For each of these subgroups, we measured associations using LPMs as in the primary analyses.

## RESULTS

The baseline sociodemographic characteristics of our 1925 participants, 960 in Uganda and 965 in Zambia, are described in Table 1. The median age of participants was 28 years (interquartile range [IQR] 24–32 years) in Uganda and 25 years (IQR 21–30 years) in Zambia. The age ranges and sociodemographic characteristics of the participants in our study are similar to those of the FSW populations described by data from key population surveillance in Uganda and Zambia.^42,43^ The majority of participants in both settings had not tested for HIV in the past 12 months (63% Uganda; 60% Zambia).

**TABLE 1.**
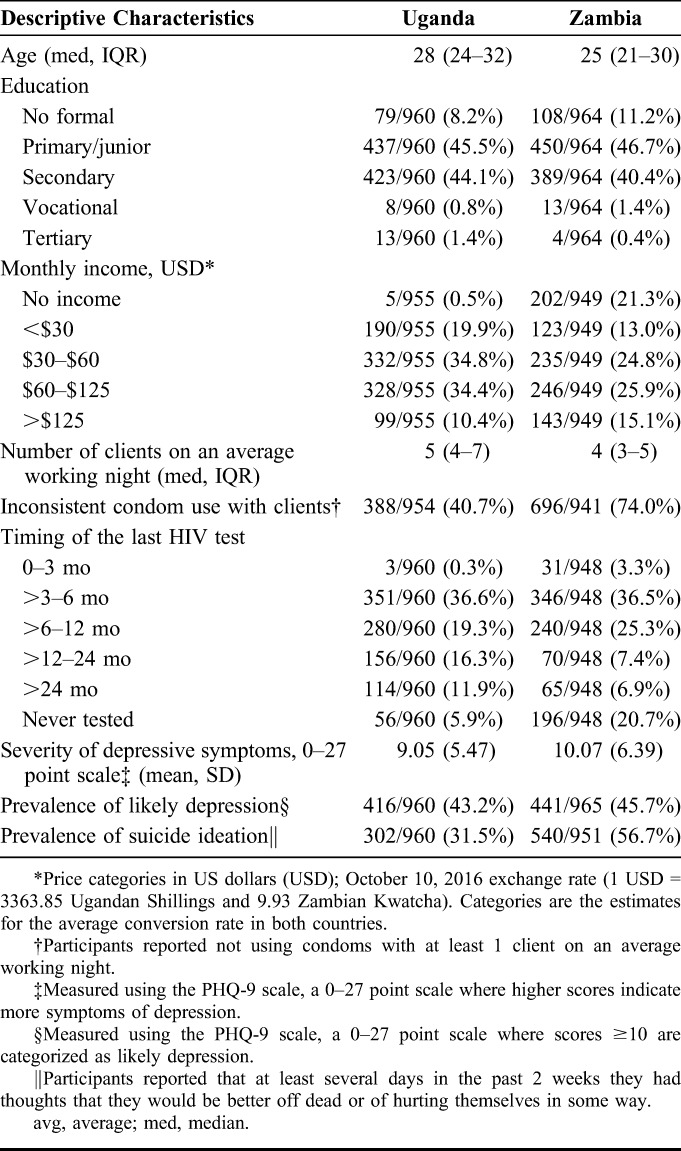
Participant Characteristics at Baseline, by Study Site

**FIGURE 1.**
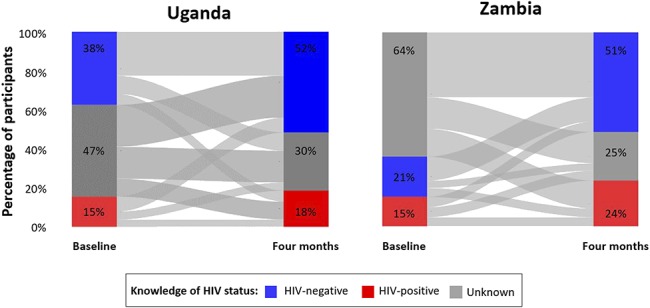
FSWs' knowledge of HIV status at baseline, 1 month, and 4 months in Uganda and Zambia. HIV-negative status knowledge (blue); HIV-positive status knowledge (red); HIV status knowledge unknown (gray). Lines between the bars show how participants changed their HIV status knowledge over the duration of the study.

The majority of participants in Uganda (57%) and in Zambia (67%) changed their knowledge of HIV status over 4 months (Fig. 1). At baseline, knowledge of HIV status was unknown among the overall majority of participants in both studies (47% Uganda; 64% Zambia), but by 4 months, knowledge of HIV status was HIV negative among just over half of participants in both settings (52% Uganda; 51% Zambia) and knowledge of HIV status was unknown among only 30% of participants in Uganda and 25% of participants in Zambia.

**FIGURE 2.**
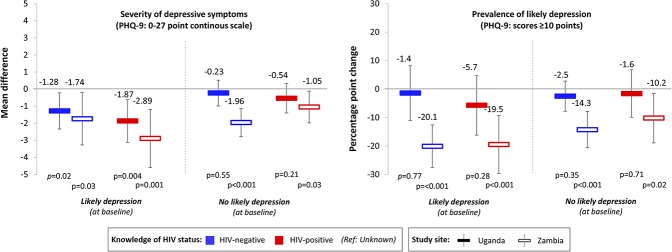
The associations between FSWs' knowledge of HIV status and depressive symptom outcomes. Associations were measured using LPMs with individual fixed effects, controlling for round of data collection and calendar month. Standard errors were clustered at the level of the peer educator. To measure depression, we used the PHQ-9 scale (0–27 points, scores ≥10 categorized as likely depression). Solid bars indicate estimates for Uganda; hollow bars indicate estimates for Zambia. Blue bars indicate knowledge of HIV-negative status; red bars indicate knowledge of HIV-positive status (reference category: unknown HIV status). Black vertical lines indicate 95% confidence intervals.

Knowledge of any HIV status was associated with significant reductions in the severity of depressive symptoms among participants in both Uganda and Zambia (Fig. 2). Compared with unknown HIV status, knowledge of HIV-negative status was associated with a 1.06-point decrease (95% CI −1.79 to −0.34, *P* = 0.004) in the severity of participants' depressive symptoms (27-point scale) in Uganda (from a baseline mean of 9.05 points) and a 1.68-point decrease (95% CI −2.70 to −0.62, *P* = 0.002) in the severity of participants' depressive symptoms in Zambia (from a baseline mean of 10.07 points). Similarly, knowledge of HIV-positive status was associated with a 1.01-point decrease (95% CI: −1.82 to −0.20, *P* = 0.02) in the severity of participants' depressive symptoms in Uganda and a 1.98-point decrease (95% CI: −3.09 to −0.88, *P* = 0.001) in the severity of participants' depressive symptoms in Zambia.

Knowledge of any HIV status was not associated with the prevalence of likely depression among participants in Uganda but was associated with significant reductions in the prevalence of likely depression among participants in Zambia. Compared with unknown HIV status, knowledge of HIV-negative status was associated with a 14.1% decrease (95% CI: −22.1% to −6.0%, *P* = 0.001) and knowledge of HIV-positive status was associated with a 14.3% decrease (95% CI: −23.9% to −4.5%, *P* = 0.002) in participant's prevalence of likely depression (PHQ-9 score ≥10), from a 45.7% prevalence of likely depression at enrollment.

**FIGURE 3.**
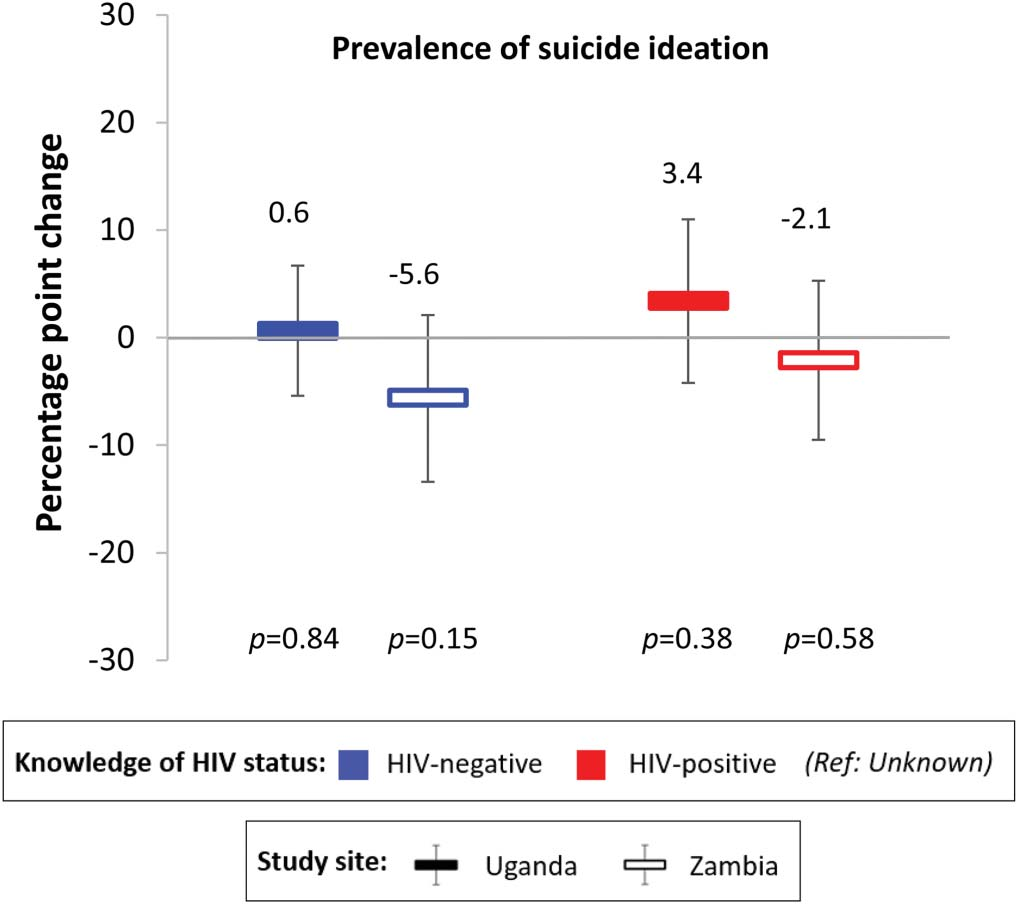
The associations between FSWs' knowledge of HIV status and prevalence of suicidal ideation. Associations were measured using LPMs with individual fixed effects, controlling for round of data collection and calendar month. Standard errors were clustered at the level of the peer educator. Suicidal ideation was measured as a component of the PHQ-9 depression scale. Participants reported that at least several days in the past 2 weeks they had thoughts that they would be better off dead or of hurting themselves in some way. Solid bars indicate estimates for Uganda; hollow bars indicate estimates for Zambia. Blue bars indicate knowledge of HIV-negative status; red bars indicate knowledge of HIV-positive status (reference category: unknown HIV status). Black vertical lines indicate 95% confidence intervals.

Knowledge of HIV status was not associated with the prevalence of suicidal ideation among participants in both Ugandan and Zambian (Fig. 3). All findings remained essentially the same in our 3 sensitivity analyses (see Table 1, Supplemental Digital Content, http://links.lww.com/QAI/B390).

**FIGURE 4.**
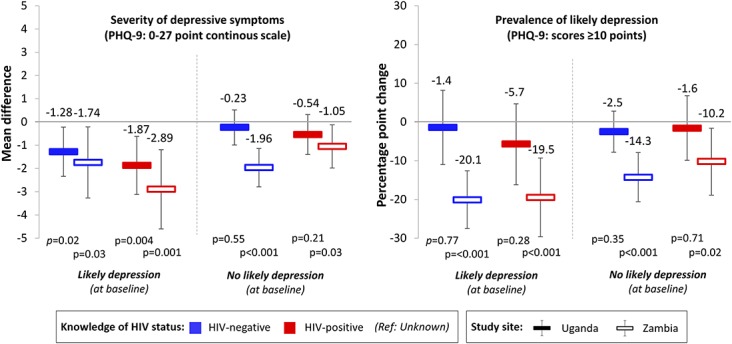
The associations between FSWs' knowledge of HIV status and depressive symptom outcomes, by those with and without likely depression at baseline. Associations were measured using LPMs with individual fixed effects, controlling for round of data collection and calendar month. Standard errors were clustered at the level of the peer educator. To measure depression we used the PHQ-9 scale (0–27 points, scores ≥10 categorized as likely depression). Solid bars indicate estimates for Uganda; hollow bars indicate estimates for Zambia. Blue bars indicate knowledge of HIV-negative status; red bars indicate knowledge of HIV-positive status (reference category: unknown HIV status). Black vertical lines indicate 95% confidence intervals.

Our subgroup analyses by the prevalence of likely depression at baseline and randomization arm, however, uncovered some novel findings. In our subgroup analyses by the prevalence of likely depression at baseline, we found greater decreases in the associations between knowledge of HIV status and both the severity of depressive symptoms as well as the prevalence of likely depression among participants who had likely depression at baseline in Zambia (Fig. 4). None of the associations between knowledge of HIV status and the severity of depressive symptoms or prevalence of likely depression was significant among participants without likely depression at baseline in Uganda. In our subgroup analyses by randomization arm, we found that knowledge of HIV-positive status was not associated with a significant decrease in depressive symptoms for participants randomized to standard-of-care HIV testing services in Uganda (mean difference: −15.3 points, 95% CI −3.20 to 0.13, *P* = 0.07) and in Zambia (mean difference: −0.45 points, 95% CI: −2.11 to 1.19, *P* = 0.58) (see Table 2, Supplemental Digital Content, http://links.lww.com/QAI/B390). Knowledge of HIV-positive status was also not associated with a decrease in the prevalence of likely depression among participants randomized to standard-of-care testing services in Zambia (percentage point change: −8.2%, 95% CI −25.0% to 8.7%, *P* = 0.34).

## DISCUSSION

Knowledge of HIV status, be it HIV-negative status or HIV-positive status, seemed to be associated with significant reductions in the severity of depressive symptoms among 2 diverse populations of FSWs compared with unknown HIV status. In Zambia, but not Uganda, knowledge of HIV status was also significantly associated with reductions the prevalence of likely depression among FSWs. Knowledge of HIV status was not associated with FSWs' suicidal ideations in either setting.

Our finding that knowledge of HIV-negative status is associated with reductions in FSWs' severity of depressive symptoms is consistent with theories of human emotion^44^ and the extant literature.^45,46^ By contrast, our finding that knowledge of HIV-positive status is associated with reductions in FSWs' severity of depressive symptoms is contrary to the prevalent hypothesis (and indeed our hypothesis) that learning of a positive HIV diagnosis would trigger fear of the physical and social consequences of HIV and induce depression and potentially suicidal thoughts.^10,11,12,13,14,15,16,17^ This pathway from HIV diagnosis to depression and suicide is a commonly cited concern with HIV testing interventions that move testing outside the health care system where counseling is provided (eg, self-testing, door-to-door testing).^8,9^

Our findings are consistent, however, with the literature in psychology around the stress of uncertainty and the benefits of knowledge, even if it brings unwanted results.^47,48^ Knowledge of HIV-positive status may be associated with reductions in FSWs' depressive symptoms for a variety of reasons. First, as discussed, it may reduce uncertainty, which can be stressful.^47,48^ Studies among cancer patients have shown a strong preference for information about their disease and its treatment, even if the information is bad.^49^ FSWs in both of these populations were frequently putting themselves at great risk of HIV infection, and thus, it is possible they felt relieved when they learned they were living with HIV and no longer had to fear infection. Second, certain knowledge of HIV-positive status might make FSWs feel empowered to take actions against the effects of HIV and control of their health.^50,51,52^ Knowledge of HIV-positive status may thus lead to an emotional boost because it enhances self-efficacy and increases the ability to act. Third, knowledge of HIV-positive status may lead FSWs to seek more information about HIV treatment. As a result, they may learn that HIV treatment is highly effective and that HIV infection no longer needs to lead to substantial morbidity and mortality,^53^ as long as treatment is available and taken consistently. This new-found or deepened knowledge may lead to a persistent, improved emotional state compared with the state of uncertain knowledge of HIV status.

Our subgroup analyses highlighted significantly greater associations between knowledge of HIV status and depressive symptoms among participants with likely depression at baseline in Zambia and no significant associations among participants without likely depression at baseline in Uganda. This finding suggests that the benefits of knowledge of HIV status on depressive symptoms might be greatest among individuals who are already depressed and can thus experience a substantial improvement in the severity of their depressive symptoms. In addition, our subgroup analyses found that the significant negative association between knowledge of HIV-positive status and the severity of depressive symptoms or prevalence of likely depression did not hold true for participants randomized to standard-of-care HIV testing services. This finding suggests that how participants change their knowledge of HIV status—for example, through HIV self-testing rather than facility-based testing—might modify the association between knowledge of HIV status and depressive symptoms. For example, with HIV self-testing, participants can maintain their privacy when they learn they are living with HIV and can freely choose who to disclose their HIV-positive status to, which may influence their depressive symptoms.

The magnitude of the associations between knowledge of HIV status and depressive symptoms also differed in the 2 study settings, with the associations being greater in Zambia compared with Uganda. The differences in associations between knowledge of HIV status and depressive symptoms by the study site might be attributable to differential access to FSW-friendly health services in these areas. In Kampala, FSWs have access to numerous free health services, including HIV testing, that are tailored to their needs and demands through MARPI health care facilities. By contrast, FSWs in the 3 Zambian transit towns have to use standard public health care facilities where they might face stigma and discrimination from health care providers. In Zambia, a larger proportion of FSWs—roughly 1 in 5—had never tested for HIV than in Uganda. As a result, knowledge of HIV status might have been more novel and longer awaited among FSWs in Zambia compared with FSWs in Uganda, resulting in a greater decrease of depressive symptoms.

To maintain an updated knowledge of HIV status and gain the mental health benefits associated with this, FSWs need to test for HIV frequently (ie, every 3 months, as recommended by the World Health Organization^54,55^). FSWs' knowledge, or perceived knowledge, of HIV status may change repeatedly because they often have new HIV risk encounters with clients as a result of strong economic incentives for condomless sex. FSWs may receive double to triple the amount of income for sex without a condom compared with sex with a condom.^56,57^ In addition, FSWs are likely to suffer from intimate partner violence and sexual abuse, which further increase their risk of HIV infection.^58,59,60^ Despite this high HIV risk, FSWs in many communities do not test or only test for HIV infrequently for reasons including physical and financial barriers to testing^61,62^ and the fear of provider stigma.^27,62,63^ Novel approaches of HIV testing interventions are thus needed, which make testing accessible and easy for members of this population.

Our study has a number of strengths. First, our study had little loss to follow-up, which reduces the potential for attrition biases. Second, the use of 2 cohorts in geographically diverse settings strengthens the generalizability of our results. Third, the use of individual fixed-effects panel estimation is a rigorous quasi-experimental method for causal inference,^24^ especially because the majority of participants in both cohorts changed their knowledge of HIV status over 4 months. Individual fixed effects control for all baseline and other time-invariant characteristics, including those that are unobserved (eg, psychological characteristics that are likely to affect the prevalence of depressive symptoms) and thus cannot be controlled for as covariates in our analyses.^24,41^

An important limitation of our fixed-effects estimation, however, is that we cannot rule out those time-varying confounders that might have biased our results. For example, from baseline to 4 months, all participants received support from peer educators, which may have influenced both their knowledge of HIV status (through HIV testing) and severity of depressive symptoms. With our approach, we are also unable to measure precisely when knowledge of HIV status changed. Although our window of observation was relatively short (4 months), it is possible that changes in knowledge of HIV status has an acute effect on depression, which we are unable to disentangle from the effect of depression over 4 months. Although our results substantially strengthen the evidence on the effect of HIV status knowledge on depressive symptoms among FSWs, they do not allow for the same strength of causal interpretation as a randomized controlled trial.

Another limitation of this study is that the PHQ-9 depression is a screening tool for depression and not a true diagnosis of clinical depression. Like all self-reported outcomes, our measurements of depression are subject to social desirability bias. The cutoff of ≥10-points that we used for a classification of likely depression is most commonly used in the literature (sensitivity of 85% and specificity of 89%^30^) and has been previously validated in SSA but is still an imperfect indicator of depression as compared with clinical diagnosis. Measuring clinical depression is time-intensive and expensive and was not feasible within the context of these trials. Importantly, our results were robust to analyses with continuous PHQ-9 scores and sensitivity analysis using different model specifications, subsets of participants, and covariates. Also, some symptoms of depression on the PHQ-9 depression scale may be symptoms of early HIV treatment side effects (ie, poor appetite and overeating, difficulty in sleeping).^64^ If our depression scale is picking up symptoms of early HIV treatment initiation, this would bias our estimates of depression at 4 months upward and consequently bias the magnitude of our effect size estimates downward. However, we expect this potential mechanism to affect a small share of our sample because the percentage of participants who had initiated HIV treatment by 4 months in both trials was low.^3,4^

Our study has important policy implications. First and foremost, our findings indicate that worries that FSWs who learn their HIV status are at increased risk of depression and suicide are not warranted. Increasing access to HIV testing will not only provide FSWs with the knowledge of HIV status that is needed for treatment and prevention but also reduce depressive symptoms and suicidal ideation among this vulnerable population. Thus, our findings provide an additional strong argument for expanded access to HIV testing for FSWs in SSA. Second, the boost to emotional well-being that is associated with gains in knowledge of HIV status could be leveraged for interventions that bring about positive life changes for FSWs—for example, economic empowerment,^65^ structural change,^66^ and community mobilization interventions.^67^ Policy makers should consider integrating such interventions into HIV testing services for FSWs. Third, our findings suggest that policy makers should increase access to novel HIV testing approaches that decouple HIV testing activities from the physical facilities, where counseling is provided, because these approaches are safe and can even lead to improved emotional well-being, with potentially powerful downstream effects on life and livelihood.^68,69^ An often-cited concern with HIV testing outside the facility-based health care system is that the detection and treatment of depressive symptoms and suicidal ideation is not immediately available. The findings in our subgroup analysis suggest that this concern is not valid and that HIV testing outside the health care facility is even more strongly associated with decreased depressive symptoms than testing in the health care facility.

HIV testing outside the facility-based health care system is gaining traction with several important testing options. For instance, HIV self-testing allows individuals to test in their own time and space and has been considered an important approach to reach populations that are not using facilities for HIV testing,^8,9^ such as FSWs,^27^ adolescents,^70^ and men.^70^ Door-to-door testing is an important option to provide rural and remote populations with HIV testing,^71,72^ but the health workers providing door-to-door testing (eg, community health workers or home-based carers) typically lack the skills to diagnose and treat depressive symptoms. Our findings suggest that such approaches will be safe. However, they do not mean that these approaches to testing should not be accompanied by supportive interventions facilitating access to care and social support. Indeed, the potential for social support through the peer delivery system in our study may have had an important effect, which we could not directly determine in this analysis.^73^

This study lays out an important future research agenda. First, more research should be conducted to understand the mechanisms through which changes in knowledge of HIV status affect changes in depressive symptoms. Second, the association between knowledge of HIV status and sexual behaviors should be explored in other populations besides FSWs to see whether findings are similar. Third, future studies should develop and test innovative HIV testing and counseling approaches that can reach FSWs who currently cannot or do not wish to use such services. Fourth, design research should be used to develop interventions that harness the reductions in depressive symptoms after changes in knowledge of HIV status to bring about beneficial life changes, such as replacing sex work with work that is safer and more sustainable over the life course.

## CONCLUSIONS

Much of the focus of HIV testing to date has been on identifying new individuals living with HIV^28^ so that they can initiate HIV treatment to preserve their own health^53^ and prevent HIV transmission to others.^74,75^ This study highlights the added benefit that knowledge of HIV status—irrespective of the status itself—has on the overall mental well-being of FSWs in high HIV prevalence settings, while diminishing concerns that HIV testing in the absence of support from HIV counselors may result in increased depressive symptoms and suicidal ideation. The findings from this study support the expansion of inventions that may increase the frequency of HIV testing among FSWs, moving the HIV testing activities outside the physical health care system.

## References

[1] Baral S Beyrer C Muessig K Burden of HIV among female sex workers in low-income and middle-income countries: a systematic review and meta-analysis. Lancet Infect Dis. 2012;12:538–549. 22424777 10.1016/S1473-3099(12)70066-X

[2] Shannon K Strathdee SA Goldenberg SM Global epidemiology of HIV among female sex workers: influence of structural determinants. Lancet. 2015;385:55–71. 25059947 10.1016/S0140-6736(14)60931-4 PMC4297548

[3] Ortblad K Kibuuka Musoke D Ngabirano T Direct provision versus facility collection of HIV self-tests among female sex workers in Uganda: a cluster-randomized controlled health systems trial. PLoS Medicine. 2017;14:e1002458. 29182634 10.1371/journal.pmed.1002458 PMC5705079

[4] Chanda M Ortblad K Mwale M HIV self-testing among female sex workers in Zambia: a cluster randomized controlled trial. PLoS Medicine. 2017;14:e1002442. 29161260 10.1371/journal.pmed.1002442 PMC5697803

[5] Masters SH Agot K Obonyo B Promoting partner testing and couples testing through secondary distribution of HIV self-tests: a randomized clinical trial. PLoS Medicine. 2016;13:e1002166. 27824882 10.1371/journal.pmed.1002166 PMC5100966

[6] Jamil MS Prestage G Fairley CK Effect of availability of HIV self-testing on HIV testing frequency in gay and bisexual men at high risk of infection (FORTH): a waiting-list randomised controlled trial. Lancet HIV. 2017;4:e241–e250. 28219619 10.1016/S2352-3018(17)30023-1

[7] Johnson CC Kennedy C Fonner V Examining the effects of HIV self-testing compared to standard HIV testing services: a systematic review and meta-analysis. J Int AIDS Soc. 2017;20:21594. 28530049 10.7448/IAS.20.1.21594 PMC5515051

[8] Wood BR Ballenger C Stekler JD Arguments for and against HIV self-testing. HIV AIDS (Auckl). 2014;6:117–126. 25114592 10.2147/HIV.S49083 PMC4126574

[9] Myers JE El-sadr WM Zerbe A Rapid HIV self-testing: long in coming but opportunities beckon. AIDS. 2013;27:1687–1695. 23807269 10.1097/QAD.0b013e32835fd7a0

[10] Ciesla JA Roberts JE Meta-analysis of the relationship between HIV infection and risk for depressive disorders. Am J Psychiatry. 2001;158:725–730. 11329393 10.1176/appi.ajp.158.5.725

[11] Kiene SM Lule H Sileo KM Depression, alcohol use, and intimate partner violence among outpatients in rural Uganda: vulnerabilities for HIV, STIs and high risk sexual behavior. BMC Infect Dis. 2017;17:88. 28103834 10.1186/s12879-016-2162-2 PMC5248514

[12] Kaharuza FM Bunnell R Moss S Depression and CD4 cell count among persons with HIV infection in Uganda. AIDS Behav. 2006;10(4 suppl):S105–S111. 16802195 10.1007/s10461-006-9142-2

[13] Monahan PO Shacham E Reece M Validity/reliability of PHQ-9 and PHQ-2 depression scales among adults living with HIV/AIDS in western Kenya. J Gen Intern Med. 2009;24:189–197. 19031037 10.1007/s11606-008-0846-z PMC2629000

[14] Nakasujja N Skolasky RL Musisi S Depression symptoms and cognitive function among individuals with advanced HIV infection initiating HAART in Uganda. BMC Psychiatry. 2010;10:44. 20537129 10.1186/1471-244X-10-44 PMC2901316

[15] Tomlinson M Grimsrud AT Stein DJ The epidemiology of major depression in South Africa: results from the South African Stress and Health study. S Afr Med J. 2009;99 Available at: https://www.ajol.info/index.php/samj/article/view/50768. Accessed June 12, 2018. PMC3195337 19588800

[16] Ovuga E Boardman J Wasserman D The prevalence of depression in two districts of Uganda. Soc Psychiatry Psychiatr Epidemiol. 2005;40:439–445. 16003593 10.1007/s00127-005-0915-0

[17] Mayston R Kinyanda E Chishinga N Mental disorder and the outcome of HIV/AIDS in low-income and middle-income countries: a systematic review. AIDS. 2012;26(suppl 2):S117–S135. 23303434 10.1097/QAD.0b013e32835bde0f

[18] Prince M Patel V Saxena S No health without mental health. Lancet. 2007;370:859–877. 17804063 10.1016/S0140-6736(07)61238-0

[19] Smit J Myer L Middelkoop K Mental health and sexual risk behaviours in a South African township: a community-based cross-sectional study. Public Health. 2006;120:534–542. 16684549 10.1016/j.puhe.2006.01.009

[20] Agardh A Cantor-Graae E Ostergren P-O Youth, sexual risk-taking behavior, and mental health: a study of university students in Uganda. Int J Behav Med. 2012;19:208–216. 21590465 10.1007/s12529-011-9159-4 PMC3358553

[21] Nduna M Jewkes RK Dunkle KL Associations between depressive symptoms, sexual behaviour and relationship characteristics: a prospective cohort study of young women and men in the Eastern Cape, South Africa. J Int AIDS Soc. 2010;13:44. 21078150 10.1186/1758-2652-13-44 PMC2992477

[22] Musisi S Wagner GJ Ghosh-Dastidar B Depression and sexual risk behaviour among clients about to start HIV antiretroviral therapy in Uganda. Int J STD AIDS. 2014;25:130–137. 23970636 10.1177/0956462413495186 PMC3841244

[23] Rogers MR Lemstra ME Moraros JS Risk indicators of depressed mood among sex-trade workers and implications for HIV risk behaviour. Can J Psychiatry Rev Can Psychiatr. 2015;60:548–555. 10.1177/070674371506001205 PMC4679163 26720823

[24] Bärnighausen T Oldenburg C Tugwell P Quasi-experimental study designs series-paper 7: assessing the assumptions. J Clin Epidemiol. 2017;89:53–66. 28365306 10.1016/j.jclinepi.2017.02.017

[25] Gunasekara FI Richardson K Carter K Fixed effects analysis of repeated measures data. Int J Epidemiol. 2014;43:264–269. 24366487 10.1093/ije/dyt221

[26] CDC. Crane survey: select results from recent surveys, Kampala 2012/3. Presented at the: Kampala, Uganda. Available at: http://documentslide.com/documents/crane-survey-select-results-from-recent-surveys-kampala-20123-men-who-have.html. Accessed March 28, 2017.

[27] Chanda M Perez-Brumer A Ortblad K Barriers and facilitators to HIV testing among Zambia female sex workers in three transit hubs. AIDS Patient Care STDs. 2017;31:290–296. 28581820 10.1089/apc.2017.0016 PMC5512327

[28] UNAIDS. Ending AIDS: Progress towards the 90-90-90 Targets. Geneva, Switzerland: UNAIDS; 2017 Available at: http://www.unaids.org/sites/default/files/media_asset/Global_AIDS_update_2017_en.pdf. Accessed August 1, 2017.

[29] Kroenke K Spitzer RL The PHQ-9: a new depression diagnostic and severity measure. Psychiatr Ann. 2002;32:509–515.

[30] Manea L Gilbody S McMillan D Optimal cut-off score for diagnosing depression with the Patient Health Questionnaire (PHQ-9): a meta-analysis. CMAJ Can Med Assoc J. 2012;184:E191–E196. 22184363 10.1503/cmaj.110829 PMC3281183

[31] Bhana A Rathod SD Selohilwe O The validity of the Patient Health Questionnaire for screening depression in chronic care patients in primary health care in South Africa. BMC Psychiatry. 2015;15:118. 26001915 10.1186/s12888-015-0503-0 PMC4446842

[32] Gelaye B Williams MA Lemma S Validity of the patient health questionnaire-9 for depression screening and diagnosis in east Africa. Psychiatry Res. 2013;210:653–661. 23972787 10.1016/j.psychres.2013.07.015 PMC3818385

[33] Ali G-C Ryan G De Silva MJ Validated screening tools for common mental disorders in low and middle income countries: a systematic review. PLoS One. 2016;11:e0156939. 27310297 10.1371/journal.pone.0156939 PMC4911088

[34] Cholera R Gaynes BN Pence BW Validity of the Patient Health Questionnaire-9 to screen for depression in a high-HIV burden primary healthcare clinic in Johannesburg, South Africa. J Affect Disord. 2014;167:160–166. 24972364 10.1016/j.jad.2014.06.003 PMC4264106

[35] Chibanda D Verhey R Gibson LJ Validation of screening tools for depression and anxiety disorders in a primary care population with high HIV prevalence in Zimbabwe. J Affect Disord. 2016;198:50–55. 27011359 10.1016/j.jad.2016.03.006

[36] Tavakol M Dennick R Making sense of Cronbach's alpha. Int J Med Educ. 2011;2:53–55. 28029643 10.5116/ijme.4dfb.8dfd PMC4205511

[37] Hartmann M McConnell M Bekker L-G Motivated reasoning and HIV risk? Views on relationships, trust, and risk from young women in Cape Town, South Africa, and implications for oral PrEP. AIDS Behav. 2018;22:3468–3479. 29404757 10.1007/s10461-018-2044-2 PMC6077112

[38] Wringe A Roura M Urassa M Doubts, denial and divine intervention: understanding delayed attendance and poor retention rates at a HIV treatment programme in rural Tanzania. AIDS Care. 2009;21:632–637. 19444672 10.1080/09540120802385629

[39] Asgary R Antony S Grigoryan Z Community perception, misconception, and discord regarding prevention and treatment of infection with human immunodeficiency virus in Addis Ababa, Ethiopia. Am J Trop Med Hyg. 2014;90:153–159. 24218413 10.4269/ajtmh.13-0215 PMC3886413

[40] Ortblad KF Musoke DK Ngabirano T Is knowledge of HIV status associated with sexual behaviours? A fixed effects analysis of a female sex worker cohort in urban Uganda. J Int AIDS Soc. 2019;22:e25336. 31287625 10.1002/jia2.25336 PMC6615530

[41] Baltagi B Econometric Analysis of Panel Data. 5th ed Chichester, United Kingdom: John Wiley & Sons, Inc; 2014.

[42] Doshi RH Sande E Ogwal M Progress toward UNAIDS 90-90-90 targets: a respondent-driven survey among female sex workers in Kampala, Uganda. PLoS One. 2018;13:e0201352. 30231030 10.1371/journal.pone.0201352 PMC6145590

[43] Behavioral and Biologic Surveillance Survey Zambia—Female Sex Workers. Family Health International, Zambia National AIDS Council; 2000 Available at: https://www.fhi360.org/sites/default/files/media/documents/Behavioral%20and%20Biologic%20Surveillance%20Survey%20Female%20Sex%20Workers.pdf. Accessed May 6, 2019.

[44] Argyle M Is happiness a cause of health? Psychol Health. 1997;12:769–781.

[45] Nease DE Aikens JE Klinkman MS Toward a more comprehensive assessment of depression remission: the Remission Evaluation and Mood Inventory Tool (REMIT). Gen Hosp Psychiatry. 2011;33:279–286. 21601725 10.1016/j.genhosppsych.2011.03.002

[46] Aikens JE Klinkman MS Sen A Improving the assessment of depression remission with the remission evaluation and mood inventory tool. Int J Psychiatry Med. 2015;50:383–397. 26526397 10.1177/0091217415612734

[47] Peters A McEwen BS Friston K Uncertainty and stress: why it causes diseases and how it is mastered by the brain. Prog Neurobiol. 2017;156:164–188. 28576664 10.1016/j.pneurobio.2017.05.004

[48] de Berker AO Rutledge RB Mathys C Computations of uncertainty mediate acute stress responses in humans. Nat Commun. 2016;7:10996. 27020312 10.1038/ncomms10996 PMC4820542

[49] Fallowfield L Ford S Lewis S No news is not good news: information preferences of patients with cancer. Psychooncology. 1995;4:197–202. 11655006 10.1002/pon.2960040305

[50] Wall KM Kilembe W Vwalika B Sustained effect of couples' HIV counselling and testing on risk reduction among Zambian HIV serodiscordant couples. Sex Transm Infect. 2017;93:259–266. 28082662 10.1136/sextrans-2016-052743 PMC5520263

[51] Rosenberg NE Pettifor AE De Bruyn G HIV testing and counseling leads to immediate consistent condom use among South African stable HIV-discordant couples. J Acquir Immune Defic Syndr. 2013;62:226–233. 23117500 10.1097/QAI.0b013e31827971ca PMC3548982

[52] Weinhardt LS Carey MP Johnson BT Effects of HIV counseling and testing on sexual risk behavior: a meta-analytic review of published research, 1985–1997. Am J Public Health. 1999;89:1397–1405. 10474559 10.2105/ajph.89.9.1397 PMC1508752

[53] Bor J Herbst AJ Newell M-L Increases in adult life expectancy in rural South Africa: valuing the scale-up of HIV treatment. Science. 2013;339:961–965. 23430655 10.1126/science.1230413 PMC3860268

[54] WHO. Consolidated Guidelines on HIV Testing Services; 5Cs: Consent, Confidentiality, Counseling, Correct Results and Connection. Geneva, Switzerland: WHO; 2015 Available at: http://apps.who.int/iris/bitstream/10665/179870/1/9789241508926_eng.pdf. Accessed May 6, 2019. 26378328

[55] Prevention and Treatment of HIV and Other Sexually Transmitted Infections for Sex Workers in Low- and Middle-Income Countries. Geneva, Switzerland: World Health Organization; 2012 Available at: http://apps.who.int/iris/bitstream/10665/77745/1/9789241504744_eng.pdf. Accessed August 19, 2017. 26131541

[56] Elmes J Nhongo K Ward H The price of sex: condom use and the determinants of the price of sex among female sex workers in Eastern Zimbabwe. J Infect Dis. 2014;210(suppl_2):S569–S578. 25381377 10.1093/infdis/jiu493 PMC4231645

[57] Quaife M Vickerman P Manian S The effect of HIV prevention products on incentives to supply condomless commercial sex among female sex workers in South Africa. Health Econ. 2018;27:1550–1566. 29926508 10.1002/hec.3784 PMC6175015

[58] Wilson KS Deya R Masese L Prevalence and correlates of intimate partner violence in HIV-positive women engaged in transactional sex in Mombasa, Kenya. Int J STD AIDS. 2016;27:1194–1203. 26464502 10.1177/0956462415611514 PMC4829471

[59] Wilson KS Wanje G Yuhas K A prospective study of intimate partner violence as a risk factor for detectable plasma viral load in HIV-positive women engaged in transactional sex in Mombasa, Kenya. AIDS Behav. 2016;20:2065–2077. 27142058 10.1007/s10461-016-1420-z PMC4996676

[60] Oldenburg CE Ortblad KF Chanda MM Brief report: intimate partner violence and antiretroviral therapy initiation among female sex workers newly diagnosed with HIV in Zambia: a prospective study. J Acquir Immune Defic Syndr. 2018;79:435–439. 30142141 10.1097/QAI.0000000000001841 PMC6203637

[61] UNAIDS. The Gap Report. Geneva, Switzerland: UNAIDS; 2014 Available at: http://www.unaids.org/sites/default/files/media_asset/UNAIDS_Gap_report_en.pdf. Accessed May 6, 2019.

[62] Wanyenze RK Musinguzi G Kiguli J “When they know that you are a sex worker, you will be the last person to be treated”: perceptions and experiences of female sex workers in accessing HIV services in Uganda. BMC Int Health Hum Rights. 2017;17:11. 28476153 10.1186/s12914-017-0119-1 PMC5420144

[63] Lancaster KE Cernigliaro D Zulliger R HIV care and treatment experiences among female sex workers living with HIV in sub-Saharan Africa: a systematic review. Afr J AIDS Res AJAR. 2016;15:377–386. 27974017 10.2989/16085906.2016.1255652 PMC5541376

[64] Kalichman SC Rompa D Cage M Distinguishing between overlapping somatic symptoms of depression and HIV disease in people living with HIV-AIDS. J Nerv Ment Dis. 2000;188:662–670. 11048815 10.1097/00005053-200010000-00004

[65] Mccormick D Munguti K Microfinance and behaviour change among Nairobi's commercial sex workers. Small Enterp Dev. 2003;14:56–65.

[66] Gurnani V Beattie TS Bhattacharjee P An integrated structural intervention to reduce vulnerability to HIV and sexually transmitted infections among female sex workers in Karnataka state, south India. BMC Public Health. 2011;11:755. 21962115 10.1186/1471-2458-11-755 PMC3205062

[67] Blanchard AK Mohan HL Shahmanesh M Community mobilization, empowerment and HIV prevention among female sex workers in south India. BMC Public Health. 2013;13:234. 23496972 10.1186/1471-2458-13-234 PMC3621162

[68] Egede LE Ellis C Grubaugh AL The effect of depression on self-care behaviors and quality of care in a national sample of adults with diabetes. Gen Hosp Psychiatry. 2009;31:422–427. 19703635 10.1016/j.genhosppsych.2009.06.007

[69] Patel H Ghosh S The impact of self-efficacy and depression on self-care in patients with heart failure: an integrative review. Int Arch Nurs Health Care. 2017;3:087

[70] Hatzold K Gudukeya S Mutseta MN HIV self-testing: breaking the barriers to uptake of testing among men and adolescents in sub-Saharan Africa, experiences from STAR demonstration projects in Malawi, Zambia and Zimbabwe. J Int AIDS Soc. 2019;22:e25244. 30907505 10.1002/jia2.25244 PMC6432104

[71] Mulubwa C Hensen B Phiri MM Community based distribution of oral HIV self-testing kits in Zambia: a cluster-randomised trial nested in four HPTN 071 (PopART) intervention communities. Lancet HIV. 2018;6:e81–e92. 30584047 10.1016/S2352-3018(18)30258-3 PMC6361868

[72] Suthar AB Ford N Bachanas PJ Towards universal voluntary HIV testing and counselling: a systematic review and meta-analysis of community-based approaches. PLoS Medicine. 2013;10:21001496. 10.1371/journal.pmed.1001496 PMC3742447 23966838

[73] Campbell J Burns B Natukunda S Social support through observational trial participation: evidence from a longitudinal HIV adherence study in southwest Uganda. Presented at the: Adherence; 2018; Miami, USA.

[74] Cohen MS Chen YQ McCauley M Prevention of HIV-1 infection with early antiretroviral therapy. N Engl J Med. 2011;365:493–505. 21767103 10.1056/NEJMoa1105243 PMC3200068

[75] Cohen MS Chen YQ McCauley M Antiretroviral therapy for the prevention of HIV-1 transmission. N Engl J Med. 2016;375:830–839. 27424812 10.1056/NEJMoa1600693 PMC5049503

